# High Prevalence of Self-Reported Photophobia in Adult ADHD

**DOI:** 10.3389/fneur.2014.00256

**Published:** 2014-12-10

**Authors:** J. J. Sandra Kooij, Denise Bijlenga

**Affiliations:** ^1^PsyQ Psycho-Medical Programs, Expertise Center Adult ADHD, The Hague, Netherlands

**Keywords:** adult ADHD, photophobia, photosensitivity, chronotype, sleep, circadian rhythm

## Abstract

Many adult outpatients with attention-deficit/hyperactivity disorder (ADHD) report an oversensitivity to light. We explored the link between ADHD and photophobia in an online survey (*N* = 494). Self-reported photophobia was prevalent in 69% of respondents with, and in 28% of respondents without, ADHD (symptoms). The ADHD (symptoms) group wore sunglasses longer during daytime in all seasons. Photophobia may be related to the functioning of the eyes, which mediate dopamine and melatonin production systems in the eye. In the brain, dopamine and melatonin are involved in both ADHD and circadian rhythm disturbances. Possibly, the regulation of the dopamine and melatonin systems in the eyes and in the brain are related. Despite the study’s limitations, the results are encouraging for further study on the pathophysiology of ADHD, eye functioning, and circadian rhythm disturbances.

## Introduction

From clinical experience, we learned that a substantial number of adult patients with attention-deficit/hyperactivity disorder (ADHD) wear sunglasses throughout the year, even on overcast days. Many of these patients report a sensitivity or even oversensitivity of their eyes to bright light. Besides this clinical observation of photophobia, there are a number of studies indicating a link between ADHD and other optical dysfunctions. In children with ADHD, 76% have reduced visual acuity, caused by more strabismus (cross-eyedness), subnormal stereoacuity (depth detection), convergence insufficiency, and smaller optic disks (1). Another study found a prevalence of 63% myopia or hyperopia, and in total 83% refractive errors in children with ADHD (2). In comparison, in a study among Dutch schoolchildren, the prevalence of myopia and hyperopia was 36% (3). Moreover, young adults with ADHD more often have problems with depth perception, peripheral vision, visual search, visual processing speed, and difficulties with color perception, especially in the blue spectrum, as compared to matched controls (4). Many of these eye dysfunctions are the result of, or affect, the retinal function.

From chronobiology studies, we know that there is a link between retinal function and the circadian rhythm. The intensity of the light reaching the intrinsic photosensitive retinal ganglion cells (ipRGC) in the retina is signaled directly to the suprachiasmatic nuclei (SCN) of the brain, which is then translated into information about the time of the day (5, 6). The SCN in turn orchestrates the internal biological clocks and is accountable for one’s chronotype (i.e., being a “morning” or an “evening” person). Among adults with ADHD, the majority has an (extremely) late chronotype and a delayed sleep phase (7, 8). A delayed sleep phase is, among others, marked by a delayed production of the endogenous “sleep hormone” melatonin in the evening (9). The suppression of melatonin in the morning is initiated by light reaching the retina of the eye (10). Melatonin may be suppressed later or less by the use of sunglasses during the day (11). This may cause the production of melatonin in the evening to be delayed, which in turn further delays the sleep/wake cycle. This is important because late sleep is associated with a short-sleep duration that on a chronic basis has a negative impact on health, with short sleep being associated with increased risks of chronic diseases such as obesity, diabetes, cardiovascular diseases, and cancer (12, 13). It is therefore important to study the potential role of the retinal functioning and its effect on chronotype, sleep, and ADHD symptoms.

The combination of our clinical experience and the findings in the available literature suggest that there may be an increased prevalence of visual problems and photophobia in adults with ADHD. In order to study a possible link between photophobia and ADHD, we initiated a short-online survey in a large group of adults with and without ADHD (symptoms). We also explored if a delayed sleep phase (late chronotype) was related to photophobia. Results from this study can be used as a first exploration of the possible link between ADHD symptoms, chronotype, and eye functioning in general, in adults with and without ADHD (symptoms).

## Materials and Methods

### Participants

We invited participants with and without ADHD (symptoms) from the Dutch general population to fill in our online survey, via websites of ADHD patient organizations and our social media accounts (Facebook© and Twitter©). Also, therapists from our outpatient Adult ADHD clinic invited their ADHD patients to participate in our study by emailing them the link to the survey.

### Material

The study was conducted in July 2013 and consisted of a very short (<5 min) self-composed and non-validated online survey in Dutch, using software by www.thesistools.nl (survey no longer available online). The survey consisted of the complete list of the following questions: a multiple choice question on having a diagnosed ADHD (I have a diagnosis of ADHD; I do not have a diagnosis but I do have ADHD symptoms; I do not have ADHD); an item on having photophobia apart from any migraine episodes (“My eyes are sensitive to light,” yes or no); the effect of light on the eyes if one had photophobia (unpleasant sensation in the eyes/headache or pain in the eyes/dryness of the eyes; each one answered with yes or no); if one had photophobia, the number of years one has had it; if one had photophobia, which measurements one takes (no measurements/using eye drops/wearing sunglasses; each one answered with yes or no; and a text field for open answers); the average number of hours per day in every season one wears sunglasses; and one’s chronotype (“Are you a morning or an evening person?”) that was scored on a five-point Likert scale (1 = definitely a morning type to 5 = definitely an evening type). We collected the following background information: age; gender; type; and dose of medication use (the participants typed in which type and dose of medication they used, or stated that they did not use any medication); and wearing glasses or contacts (yes or no). We also collected information on possible confounders for photophobia that are known from the literature: having an eye disease or disorder (yes or no); comorbidity (migraine/diabetes/head injury/seasonal affective disorder/bipolar depression/chronic fatigue/agoraphobia; each one answered with yes or no); and having had chemotherapy (yes or no) (14). The survey was not considered burdensome and the participants were assigned an anonymous consecutive number in the database. We followed guidelines of the Dutch Personal Data Protection Act. Our study does not fall under the Dutch Medical Research Involving Human Subjects Act and therefore did not need to undergo medical ethical review. Participants gave written informed consent by filling in the online survey.

### Analysis

We used Chi-square analysis to compare categorical variables between the groups with and without ADHD (symptoms). In order to determine if any of the main and interaction factors significantly added to the odds of photophobia, we entered the relevant explanatory variables into a single binary logistic regression with photophobia as the dependent variable. The model included the following explanatory variables (reference categories between brackets): female gender (male); age >40 years (≤40 years); ADHD group (controls); extreme evening chronotype (other chronotype); eye disease (no eye disease); contacts or glasses (no contacts or glasses); reporting migraine (no migraine); reporting SAD (no SAD); reporting chronic fatigue (no chronic fatigue); interaction effect of gender and age; interaction effect of ADHD and SAD; interaction effect of extreme evening chronotype and SAD. Because the SPSS Version 20.0 for Windows (SPSS Inc., Chicago, IL, USA) was used for all statistical analyses, and α = 0.05 was used to indicate statistical significance.

## Results

In total, 543 participants filled out the surveys, of which 49 were excluded from analysis due to incomplete surveys (i.e., because they prematurely terminated the survey session). This resulted in a total of *N* = 494 participants eligible for analysis, see Table 1. Of the participants, 30% reported to have a diagnosis of ADHD, 17% reported ADHD symptoms, and 53% had no ADHD. In Table 1, we merged the ADHD and the ADHD symptoms groups (termed ADHD group) because of their similarities concerning photophobia prevalences, in order to create a larger group and thus increase the power of the statistical tests. There were more males in the ADHD group than in the control group (27 vs. 19%, χ^2^ = 3.9, df = 1, *p* = 0.048). The control group was on average slightly older, but not significantly (36.7 vs. 38.4 years, *F* = 2.8, *p* = 0.093). The groups did differed in their prevalence of use of glasses or contact lenses (58 vs. 49%, χ^2^ = 4.0, df = 1, *p* = 0.045). Some possible confounders that were asked in the survey were very low prevalent (diabetes, chemotherapy, serious head injury, bipolar disorder, and/or agoraphobia), thus, these are reported as “Other confounding factors” in Table 1.

**Table 1:** **Demographics and photophobia results of study participants, *N* = 494**.

	Diagnosis ADHD, *n* = 149	ADHD symptoms, *n* = 82	Together: ADHD group, *n* = 231	Control group, *n* = 263	ADHD group vs. Controls	
					Value	*p*
Males, n/n valid (%)	34/147 (23)	27/82 (33)	61/229 (27)	50/261 (19)	χ^2^ = 3.9	0.048
Age, *n* = 494, mean (SD)	37.0 (9.4)	36.3 (10.2)	36.7 (9.7)	38.4 (11.7)	*F* = 2.8	0.093
Glasses or contacts, n/n valid (%)	85/147 (58)	48/82 (59)	133/229 (58)	128/261 (49)	χ^2^ = 4.0	0.045
Eye disease, n/n valid (%)	13/148 (9)	7/82 (9)	20/230 (9)	13/261 (5)	χ^2^ = 2.7	0.101
Extreme evening chronotype, n/n valid (%)	40/142 (28)	18/78 (23)	58/220 (26)	48/252 (19)	χ^2^ = 3.6	0.057
Self-reported photophobia, n/n valid (%)	100/145 (69)	55/79 (70)	155/224 (69)	70/250 (28)	χ^2^ = 80.4	0.001
Of which, n/n valid (%)						
A life-long condition	51/70 (73)	19/28 (68)	70/98 (71)	24/33 (73)	χ^2^ = 0.3	0.848
Causing unpleasant sensation in eyes	52/70 (74)	20/28 (71)	72/98 (73)	24/33 (73)	χ^2^ = 0.0	0.934
Causing headache or pain in the eyes	33/70 (47)	11/28 (39)	44/98 (45)	16/33 (48)	χ^2^ = 0.1	0.721
Causing dryness of the eyes	22/70 (31)	6/28 (21)	28/98 (29)	5/33 (15)	χ^2^ = 2.4	0.125
Number of hours wearing sunglasses, *n* = 388, mean (SD)						
In spring	2.5 (2.5)	1.8 (1.9)	2.3 (2.3)	1.2 (1.5)	*F* = 32.2	<0.001
In summer	4.2 (3.0)	3.8 (3.3)	4.0 (3.1)	3.1 (3.0)	*F* = 10.6	0.001
In autumn	1.6 (2.1)	1.0 (1.4)	1.4 (1.9)	0.7 (1.2)	*F* = 20.1	<0.001
In winter	1.2 (2.0)	0.6 (1.2)	1.0 (1.8)	0.4 (0.9)	*F* = 21.6	<0.001
Comorbidity, n/n valid (%)						
Migraine	34/137 (25)	15/72 (21)	49/209 (23)	40/244 (16)	χ^2^ = 3.5	0.060
Chronic fatigue	28/137 (20)	2/72 (3)	30/209 (14)	9/244 (4)	χ^2^ = 16.3	<0.001
(Seasonal) affective disorder	56/137 (41)	21/72 (29)	77/209 (37)	21/244 (9)	χ^2^ = 52.9	<0.001
Other confounding factors^a^	12 (8)	4 (5)	16 (7)	6 (2)	χ^2^ = 6.2	0.013
Use of ADHD medication, n/n valid (%)	95/149 (64)	4/82 (5)	99/231 (43)	3/263 (1)	χ^2^ = 130.6	<0.001

*The percentages are corrected for missing values per item*.

*^a^Other confounding factors were diabetes, chemotherapy, serious head injury, bipolar disorder, and/or agoraphobia*.

Self-reported photophobia was prevalent in 69% of participants in the ADHD group, vs. 28% in the control group (χ^2^ = 80.4; df = 1; *p* < 0.001), see Table 1. There was a tendency toward higher prevalence of extreme evening chronotype in the ADHD group (26 vs. 19%, χ^2^ = 3.6; df = 1; *p* = 0.057). Photophobia was a life-long condition in 73% of the photophobia cases, and this was not significantly different between ADHD and non-ADHD groups. Bright light caused an unpleasant sensation in the eyes in 73% of the cases, headache or pain in the eyes in 46% of cases, and dryness of the eyes in 25% of cases. The ADHD group reported to wear sunglasses 2.3 h in spring (vs. 1.2 h in non-ADHD; *p* < 0.001); 4.0 h in summer (vs. 3.1 h; *p* = 0.001), 1.4 h in autumn (vs. 0.7 h; *p* < 0.001), and 1.0 h in winter (vs. 0.4 h; *p* < 0.001), see Table 1.

We have entered all relevant explanatory variables into a single binary logistic regression model with photosensitivity as the dependent variable, see Table 2. The participants reporting any of the confounding factors for photophobia, which were diabetes, chemotherapy, serious head injury, bipolar disorder, and/or agoraphobia, were excluded from the binary logistic regression, resulting in *N* = 405 included cases. Migraine was entered in the regression as a separate covariate because of its substantial prevalence. The logistic regression revealed that the main factors for having ADHD (symptoms) (OR 6.1; *p* < 0.001), and eye disease (OR 6.2; *p* = 0.002) were related to a higher odds of photophobia. There was a tendency for higher odds of photophobia for the use of contact lenses or glasses (OR 1.6; *p* = 0.052), and having (seasonal) affective disorder (OR = 2.7, *p* = 0.069). Contrary to our expectation, extreme evening chronotype had a tendency toward a lower odds of photophobia (OR 0.6; *p* = 0.096), and migraine was not related to higher odds of having photophobia (apart from any migraine episodes; OR 1.1; *p* = 0.870). Other factors were also not significantly related to photophobia, and we did not find any interaction effects.

**Table 2:** **Binary logistic regression with photosensitivity as the dependent variable, *N* = 405**.

Explanatory variables (ref)	OR	95% CI of OR	SE	*p*	VIF
Gender: female (male)	0.99	0.18–5.43	0.87	0.989	1.05
Age >40 years (≤40 years)	1.19	0.14–9.88	1.08	0.875	1.03
ADHD group (controls)	6.06	3.50–10.50	0.28	<0.001	1.16
Extreme evening chronotype: yes (no)	0.58	0.31–1.10	0.33	0.096	1.02
Eye disease: yes (no)	6.18	1.94–19.64	0.59	0.002	1.03
Contacts or glasses: yes (no)	1.58	1.00–2.51	0.24	0.052	1.02
Migraine: yes (no)	1.05	0.58–1.92	0.31	0.870	1.06
Seasonal affective disorder (SAD): yes (no)	2.69	0.93–7.80	0.54	0.069	1.19
Chronic fatigue: yes (no)	2.35	0.84–6.58	0.53	0.104	1.20
Gender × age group	1.24	0.39–3.96	0.59	0.711	
ADHD × SAD	0.50	0.14–1.74	0.64	0.274	
Extreme evening chronotype × SAD	0.72	0.19–2.70	0.68	0.623	
Constant	0.01		1.76	0.012	

*Nagelkerke *R*^2^ = 0.319*.

*OR, odds ratio; SE, standard error; VIF, variance inflation factor*.

## Discussion

This preliminary study shows a strong relationship between photophobia and ADHD (symptoms). Also, the ADHD group more frequently reported the use of contacts and glasses, and wearing sunglasses longer during the day in every season than the non-ADHD group. The photosensitivity was not significantly related to seasonal affective disorder (SAD) in our study; however, there was a tendency. A recent study by Roecklein et al. showed that patients with SAD have a weaker pupil response to blue vs. red light as compared to healthy controls, allowing more light to reach the retina, which may be experienced as oversensitivity to light (15).

These preliminary results should be interpreted in the light of the limitations of the study. The main limitation of our study is that we used an online survey, which has some methodological disadvantages with regards to selection of participants, in which the control group may not reflect the general population and the patient group may not reflect the general ADHD population, and also regarding unknown response quality and unverifiable self-report of diseases. Also, the survey may have lacked clarity in the formulation of photophobia, other than being oversensitive to light in general. We have hypothesized that the oversensitivity to light in ADHD may have been related to a behavioral adaptation such as avoiding daylight and wearing sunglasses, which in turn may prevent entrainment of the biological clock to daylight, leading to a further delay of the already delayed circadian rhythm. In the Van Veen study, there was a prevalence of 78% delayed sleep phase in a group of adults with ADHD (7).

Contrary to our hypothesis, however, in the current study the extreme evening chronotype had a tendency toward a lower odds of photophobia. We speculate that the chronotype question may not have been specific enough: we have asked if one is a morning or an evening type, without any further explanation on the inherited circadian preference for the morning or evening. This may have biased our chronotype results. Also, we did not ask the time of the day the participants wear sunglasses. Wearing sunglasses during the afternoon and early evening prevents people from delaying their circadian rhythm. Further study investigating the use of sunglasses should take the time of the day into account. Lastly, in our survey the participants were not able to specify if they used either glasses or contact lenses, so that we were not able to analyze if there was a relationship between wearing contact lenses, which can cause corneal surface damage, and photophobia, which may be the result of this damage. Similarly, ocular damage as a result of long-term use of ophthalmic solutions with preservatives and its relationship with photophobia cannot be analyzed because we did not consider this possible relationship prior to data sampling. These possible relationships should be examined in future studies.

In all, we found a strong relationship between ADHD and photophobia, but our results deserve a more thorough investigation in a representative sample of patients and healthy controls. It is yet unclear what causes the photophobia in adults with ADHD. There may be a role for the intrinsically photosensitive retinal ganglion cells (ipRGCs), which are involved in the pupillary response to light and the sensitivity of the eye to light at the one hand, and in the synthesis of dopamine and melatonin in the retina at the other hand (16, 17). Possibly, the regulation of the dopamine and melatonin systems in the eyes and in the brain are related. The dopamine system is dysregulated in ADHD, and the melatonin onset is delayed in the majority of adults with ADHD (7, 18, 19). The functioning of the dopamine and melatonin receptors in the retina is possibly impaired as well and may be related to the pathophysiology of ADHD and circadian disturbances. The role of the ipRGCs is therefore an interesting candidate for further research in ADHD. Pupillometry may give us more insight into the effect of light on the eyes, the pupillary reflex, and the relatedness of ipRGC functioning, oversensitivity to light, and the pathophysiology of ADHD.

## Conflict of Interest Statement

The Review Editor, Eus J. W. Van Someren declares that, despite having collaborated with authors Denise Bijlenga and J. J. Sandra Kooij, the review process was handled objectively and no conflict of interest exists. Dr. J. J. Sandra Kooij reports having received lecture fees from Janssen B. V. and Lilly B. V. and unrestricted research grants from Janssen B. V. and Shire B. V.; all of these for other studies. Dr. Denise Bijlenga reports no financial interests or potential conflicts of interest.
